# Global Functional Connectivity Differences between Sleep-Like States in Urethane Anesthetized Rats Measured by fMRI

**DOI:** 10.1371/journal.pone.0155343

**Published:** 2016-05-11

**Authors:** Ekaterina Zhurakovskaya, Jaakko Paasonen, Artem Shatillo, Arto Lipponen, Raimo Salo, Rubin Aliev, Heikki Tanila, Olli Gröhn

**Affiliations:** 1 A.I. Virtanen Institute for Molecular Sciences, Department of Neurobiology, University of Eastern Finland, Kuopio, Finland; 2 Department of Control and Applied Mathematics, Moscow Institute of Physics and Technology, Moscow, Russia; 3 Institute of Theoretical and Experimental Biophysics, Puschino, Moscow Region, Russia; 4 Federal Scientific & Clinical Center for Federal Biomedical Agency of Russia, Moscow, Russia; University of Alberta, CANADA

## Abstract

Sleep is essential for nervous system functioning and sleep disorders are associated with several neurodegenerative diseases. However, the macroscale connectivity changes in brain networking during different sleep states are poorly understood. One of the hindering factors is the difficulty to combine functional connectivity investigation methods with spontaneously sleeping animals, which prevents the use of numerous preclinical animal models. Recent studies, however, have implicated that urethane anesthesia can uniquely induce different sleep-like brain states, resembling rapid eye movement (REM) and non-REM (NREM) sleep, in rodents. Therefore, the aim of this study was to assess changes in global connectivity and topology between sleep-like states in urethane anesthetized rats, using blood oxygenation level dependent (BOLD) functional magnetic resonance imaging. We detected significant changes in corticocortical (increased in NREM-like state) and corticothalamic connectivity (increased in REM-like state). Additionally, in graph analysis the modularity, the measure of functional integration in the brain, was higher in NREM-like state than in REM-like state, indicating a decrease in arousal level, as in normal sleep. The fMRI findings were supported by the supplementary electrophysiological measurements. Taken together, our results show that macroscale functional connectivity changes between sleep states can be detected robustly with resting-state fMRI in urethane anesthetized rats. Our findings pave the way for studies in animal models of neurodegenerative diseases where sleep abnormalities are often one of the first markers for the disorder development.

## Introduction

Sleep is a vital physiological process [1]. We spend about 1/3 of our life asleep, and no mammal is able to survive for a long period without sleep. Sleep consists of several states with different characteristics, usually divided into rapid eye movement (REM) and non-REM (NREM) phases. Muscle atonia and rapid eye movements are typically found during REM phase; while NREM phase is usually associated with less pronounced brain activity. Although the meaning of the sleep states is still being elucidated, there is evidence indicating a significant role of these states in memory and learning process. An important role of NREM sleep (in humans, particularly, slow wave sleep) in declarative memory consolidation is well established, but many studies have found a similar contribution of NREM to procedural memories as well. In contrast, REM sleep has been ascribed to have a role in non-declarative memories, although evidence in this regard is still rather scarce [2]. All in all, both sleep stages appear to be important for normal memory functioning.

Sleep disorders are associated with several neurological diseases, such as depression, Parkinson’s disease and Alzheimer’s disease. Often, the change of sleep patterns is observed in early stages of the disease [3]. Sleep research has traditionally focused on the mechanisms that induce sleep, while the sleep-associated changes during the sleep in the cortical networks have received less attention, even though there is increasing evidence that network level connectivity plays an important role both in memory consolidation and in early phase of many neurological diseases [4].

One of the ways to estimate the brain function at a global level during different states is to measure functional connectivity between brain regions. Functional connectivity and topology between brain regions may change abruptly in response to transition from one sleep state to another [5]. The assessment of these changes can help to evaluate the role of each state. Therefore, resting-state functional magnetic resonance imaging (rs-fMRI) [6] appears an ideal non-invasive technique for sleep studies since it allows the monitoring of whole-brain connectivity and networking with relatively high spatial and temporal resolution.

Rodents have similar sleep controlling mechanisms and subsequent neurochemical modulations compared to humans [7]. Therefore, it is not surprising that several groups have exploited animal models in sleep and insomnia studies [8,9], as more invasive approaches and better controlled experimental settings can be used compared to human studies. In addition, genetically modified animals may provide an insight into mechanism of sleep disorder studies in future [10].

To date, there are no sleep fMRI studies conducted in non-anesthetized animals due to the noise in the magnet and stress caused by restraining. In addition, non-anesthetized rats have rather short periods of REM sleep [11], which is insufficient to measure functional connectivity during the REM phase. A promising, feasible approach was offered by the observation that rats under urethane anesthesia express natural sleep-like states [12]. The sleep-like states under urethane are very similar to what is found in non-anesthetized rats [13], and the duration of REM-like periods is also longer (5–10 min) than in non-anesthetized rodents. This urethane-induced sleep model has been previously explored to investigate differences in functional connectivity in olfactory system between states [14] in spontaneously breathing animals. However, the global connectivity changes in urethane-induced sleep-like states have not been explored.

Therefore, the aim of the study was to estimate the changes in global functional connectivity, networks and topology between sleep-like states in mechanically ventilated urethane anesthetized rats under carefully controlled physiological conditions.

## Materials and Methods

### Animal preparation

All animal procedures were approved by the Animal Ethics Committee of the Provincial Government of Southern Finland, and conducted in accordance with the guidelines set by the European Commission Directive 2010/63/EEC. A total of 13 adult male Wistar rats (336 ± 14g) were used in 13 experiments. The animal preparations and fMRI protocol were similar as previously in our previous report [15].

In brief, all rats were first anesthetized with isoflurane (5% for induction and 2% for maintenance during surgery) in an N2/O2 70/30 mixture. The femoral artery and vein were cannulated for arterial blood sampling and drug administrations. Tracheostomy was made for mechanical ventilation. After surgical procedures, the anesthesia was transitioned to urethane (1000 mg/kg i.v.). Additional dose of urethane was given if found necessary based on the hind limb withdrawal and palpebral reflexes prior to the administration of muscle relaxant (1 mg/kg, pancuronium bromide, Pavulon, Organon, Oss, Netherlands). The temperature, breathing rate and heart rate were monitored during the experiment using an MR-compatible small animal monitoring system (Model 1025, Small Animal Instruments Inc., New York, NY, USA). Blood samples were obtained before and after each fMRI and EEG session. The rats were sacrificed immediately after the measurements with an administration of potassium chloride (under 5% of isoflurane) following a cervical dislocation.

### EEG protocol

Four recording screw electrodes were inserted into sensory (AP -2.0 mm, ML ±3.0 mm) and motor (AP +2.0 mm, ML ±3.0 mm) cortices on left and right hemispheres, respectively, for 3/13 rats. In addition, two similar screw electrodes were implanted above the cerebellum as ground and reference electrodes. The connector was attached to a headstage (Plexon, HST/16-TR-GR-G1), and the signal was further amplified with an AC amplifier (gain 1000), 16-Channel Extracellular Differential AC Amplifier Model 3500, A-M System, USA) and digitized at 2 kHz per channel (DT2821 series A/D board; Data Translation, Marlboro, MA, USA). The signal was bandpass-filtered between 0.3 and 3000 Hz. The data were acquired by using Sciworks 5.0 program (DataWave Technologies, Loveland, CO, USA). The recordings lasted 1–2 hours.

### fMRI protocol

Functional MRI data were acquired using the 7T Bruker Pharmascan MRI scanner with single-shot SE-EPI sequences from 10/13 rats. The coronal slice package containing 11 consecutive 1.5-mm thick slices was placed to start from the anterior side of the olfactory bulb and to end at the posterior parts of the cerebellum, and imaged for each measurement with the following parameters: TR 2 s, TE 45 ms, bandwidth 250 kHz, FOV 2.5 cm x 2.5 cm, matrix size 64 x 64 and in-plane resolution 391 μm x 391 μm. Resting-state signal was acquired for 900 to 3600 volumes.

### EEG analysis

First, the lowpass filter was applied (≤ 100 Hz) and the spectrogram of the signal was created. REM-like periods were identified by a peak in theta band and decrease in slow oscillations. NREM-like periods were characterized by high power in slow oscillation band. The lengths of REM-like periods were compared to the lengths of baseline level rises in BOLD. The Kolmogorov-Smirnov two-sample test was used to test the hypothesis that time intervals come from the same distribution.

### fMRI analysis

#### Preprocessing

Preprocessing steps for fMRI data included slice-timing correction, motion correction, Gaussian smoothing, co-registration to a reference brain, and reslicing performed using the SPM8 package software (http://www.fil.ion.ucl.ac.uk/spm/software/spm8/) and AEDES package software (http://aedes.uef.fi). In addition, fMRI time series were bandpass filtered from 0.02 to 0.15 Hz to remove the linear drift and high-frequency noise.

#### ROIs

**Fig 1:**
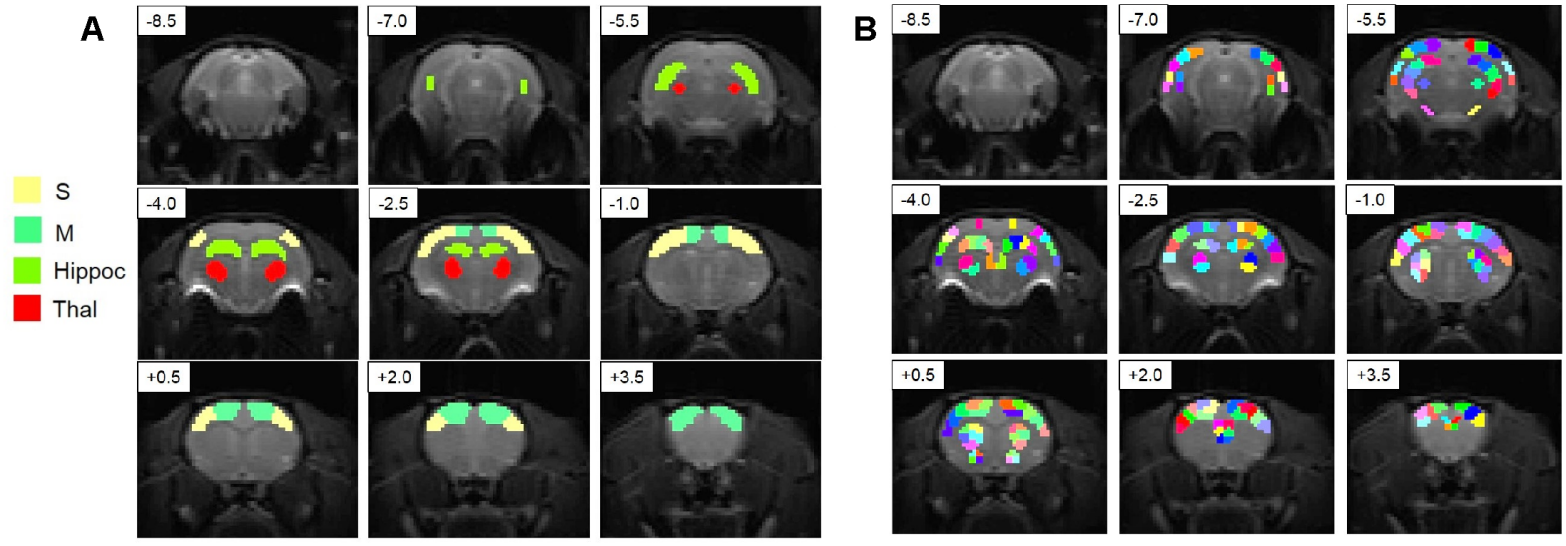
Regions of interest (ROIs) used in connectivity analysis. Slices are shown overlaid with raw EPI images. A. ROIs used for functional connectivity analysis. B. ROIs used for modularity analysis. Slices are arranged from posterior to anterior and distance from bregma in mm is given in the upper left corner of each slice. Hippoc—hippocampus, M—motor cortex, S—somatosensory cortex, Thal—thalamus.

Two sets of ROIs were drawn using a rat anatomical atlas (Fig 1). The first set of ROIs consists of eight ROIs and was used for functional connectivity analysis (Fig 1A). The second set for ROIs consisted of 171 regions and was used for topology evaluation using graph theory. The number of voxels in each region was from eight to fifteen. ROIs were covering cortical, thalamic, hippocampal and striatal regions of the brain.

For each set of ROIs the correlation matrix was created. For each region the mean time series of all voxels from that regions was calculated. Then the Pearson correlation coefficient for each pair of time series was computed resulting in N x N connectivity matrix (N is a number of ROIs).

#### Correlation map

To estimate where in the brain the transition between the sleep states takes place, we performed correlation analysis between an average state transition template and fMRI data. The state transition template was created using an average length of all detected transitions.

#### Network topology

For topology assessment, the Brain Connectivity Toolbox (https://sites.google.com/site/bctnet/Home) was used.

The networks chosen for the analysis consisted of 171 nodes (Fig 1B). First, the functional connectivity matrix was calculated. For the application of algorithms fully connected matrix was converted into a sparsely connected graph. Only connections that were above the threshold were left in the graph. As there is no gold standard for choosing a threshold, a range of connection densities from 0.1 to 0.3 was tested. The threshold was chosen so that the connection density for each graph was the same, which assures that we are comparing the structure of networks, not just the average strength of the connections.

The modularity was calculated for each graph. High modularity of the network shows that it can be subdivided in modules, where nodes inside one module are densely connected to each other but sparsely connected to nodes from other modules [16]. All graphs in the chosen range of connection densities had significantly higher modularity than randomly rewired graphs with the same degree distribution.

The values were compared between two states: REM-like and NREM-like. Results were tested for statistical significance using the Student’s t-test with FDR correction for multiple comparisons.

## Results

Resting-state fMRI or EEG signals were successfully obtained from a total of 13 urethane anesthetized rats. Clear artifacts were not detected, and all data were used in the subsequent data processing and analysis. The values analyzed from arterial blood samples indicated normal physiological condition of subjects before (pCO_2_ 39.6±5.6 mmHg, pO_2_ 142±28 mmHg, pH 7.44±0.05, sO_2_ 99.0±0.4%) and after (pCO_2_ 36.5±9.3 mmHg, pO_2_ 126±14 mmHg, pH 7.42±0.07, sO_2_ 98.9±0.5%) the fMRI and EEG measurements. Animals showed no sign of any illness that could lead to early euthanasia prior to or during the experiment.

### EEG and BOLD signals fluctuate between two sleep-like states

**Fig 2:**
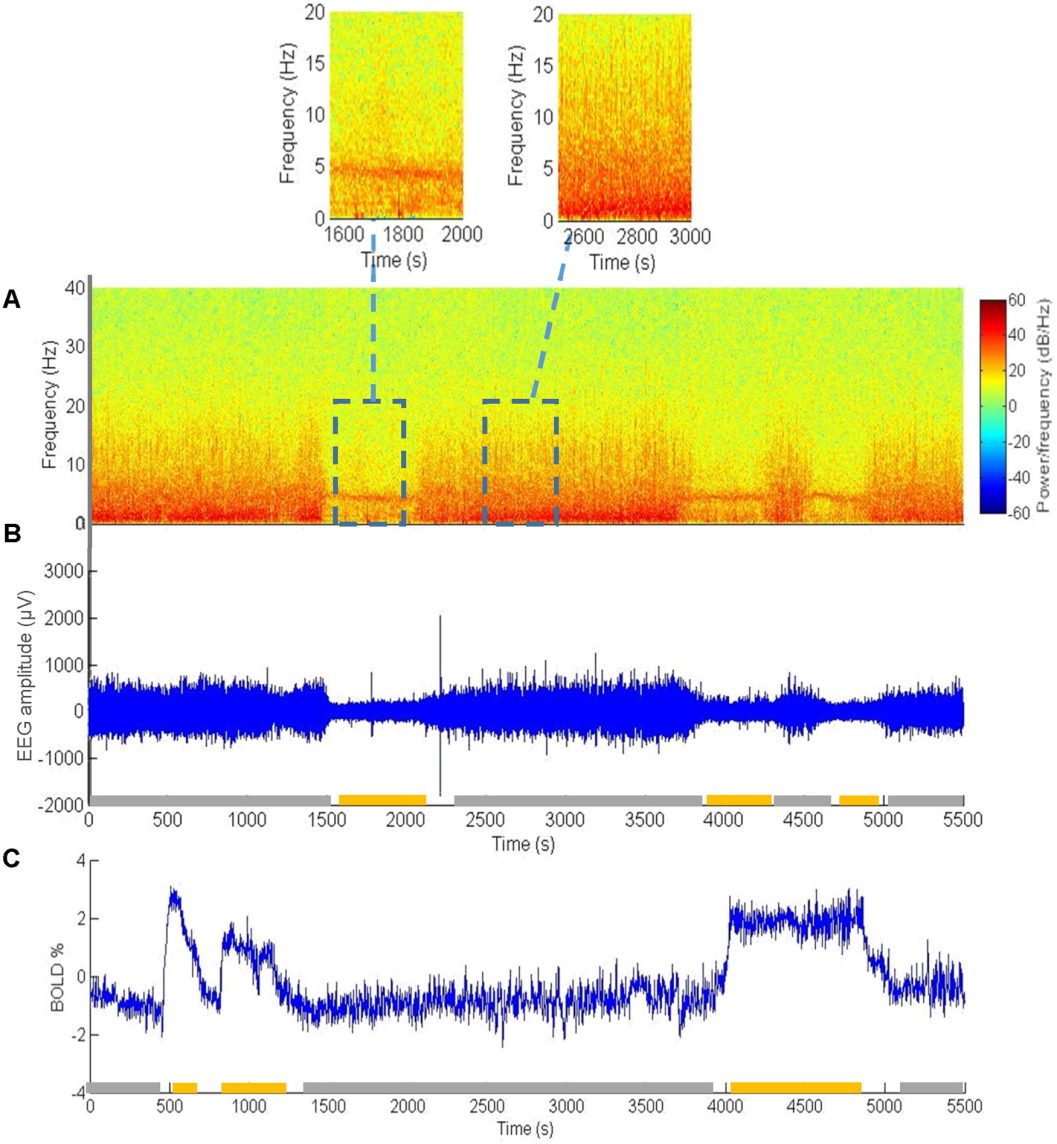
State changes in EEG and BOLD signals. Examples of EEG power spectrum (A) and bandpass filtered raw EEG signal (B) of a representative animal. The recording electrode was located in the motor cortex. Three REM-like periods are indicated by yellow horizontal bars on the x-axis. Magnified parts of signal in REM-like (left) and NREM-like (right) periods are shown in the inserts. C. An example of BOLD-fMRI data from a different animal during a similar time window. The region of interest was the motor cortex. The duration of periods with increased BOLD is similar to what was detected in EEG. In B and C the thick line above the time axis indicates the time spent in REM-like (orange) and NREM-like (gray). White spaces—transitions between states.

EEG measurements showed two clearly different states in urethane anesthetized rats (Fig 2A and 2B) attributed to REM and NREM like sleep states. REM-like states were demonstrated by power decrease in slow, delta and spindle bands and had a narrow peak in theta band when compared to NREM-like states. The theta oscillation, likely conducted from hippocampus, was used as a signature for a REM-like state. The heart rate was stable during the experiment and did not change significantly between the sleep-like states. The animals showed a regular cycling between REM-like and NREM-like states. The average durations were 5.67 ± 3.07 minutes and 23.4 ± 22.7 minutes for REM-like and NREM-like states, respectively. Correspondingly, in BOLD data measured from 10 different animals, two different baseline levels were detected lasting 6.5 ± 3.5 minutes and 24 ± 14 minutes. The duration of REM-like periods in the EEG recording and the duration of BOLD increase come from the same distribution (the hypothesis that data come from different distributions was rejected with Kolmogorov-Smirnov test, p = 0.89). Therefore, we ascertain that different BOLD baseline levels are likely to present REM and NREM like states.

### Baseline changes in BOLD signal are uniform over the brain

**Fig 3:**
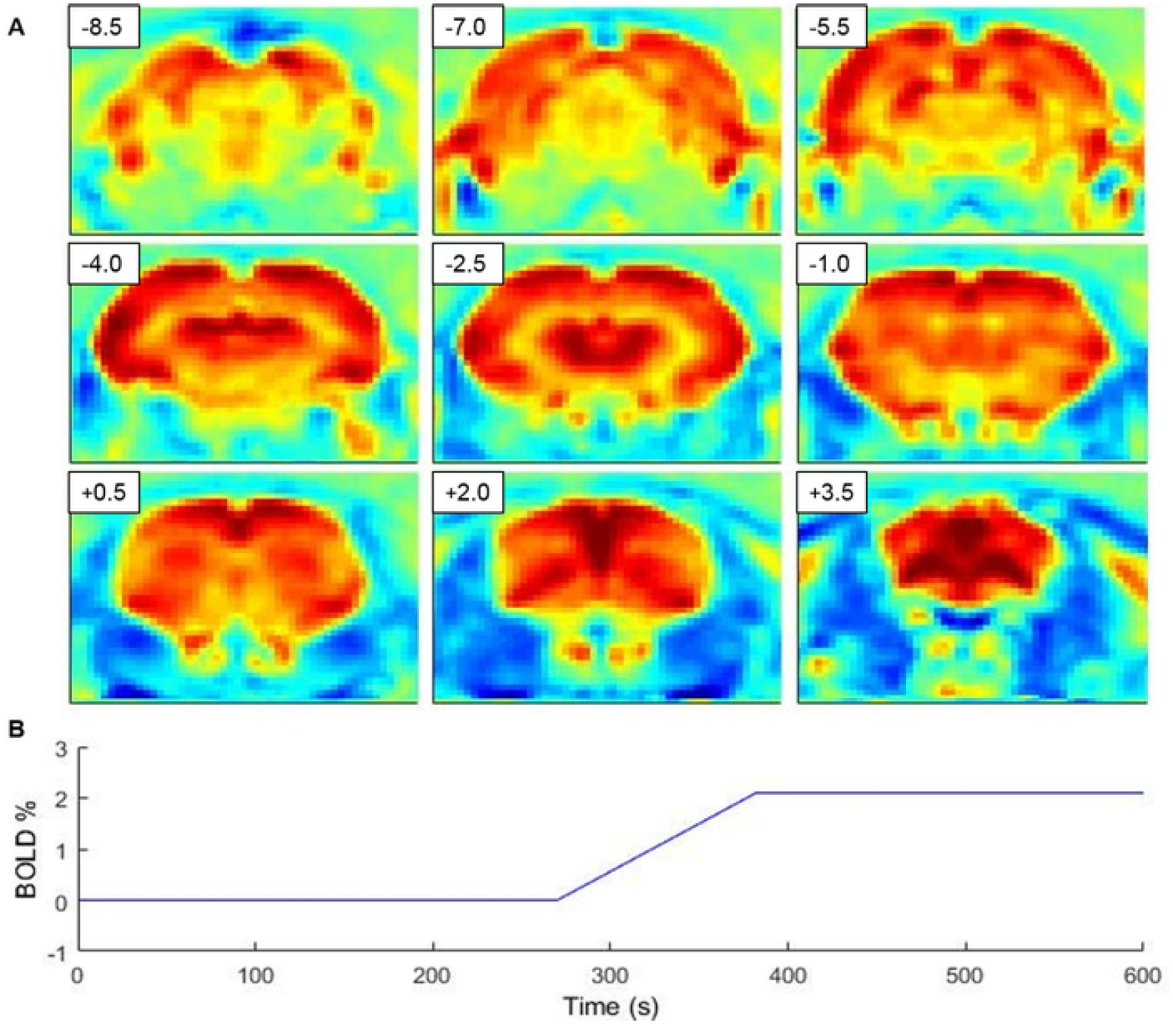
A map indicating correlation between fMRI time series and sleep state transition template. (A) demonstrates global involvement of the brain for sleep state transition. Distance from bregma in mm is given in the upper left corner of each slice. Transition template used in analysis represents the average transition length (56.9± 5.2 s) and level difference (2.1 ± 0.3 BOLD %) over all state transitions (B).

The spatial distribution of the baseline BOLD signal transitions between states was assessed using correlation analysis between the template of state changes (Fig 3B) and BOLD time series in each voxel. A correlation map shows (Fig 3A) that BOLD transition is relatively homogenous over the entire brain.

### Functional connectivity changes between the sleep-like states

**Fig 4:**
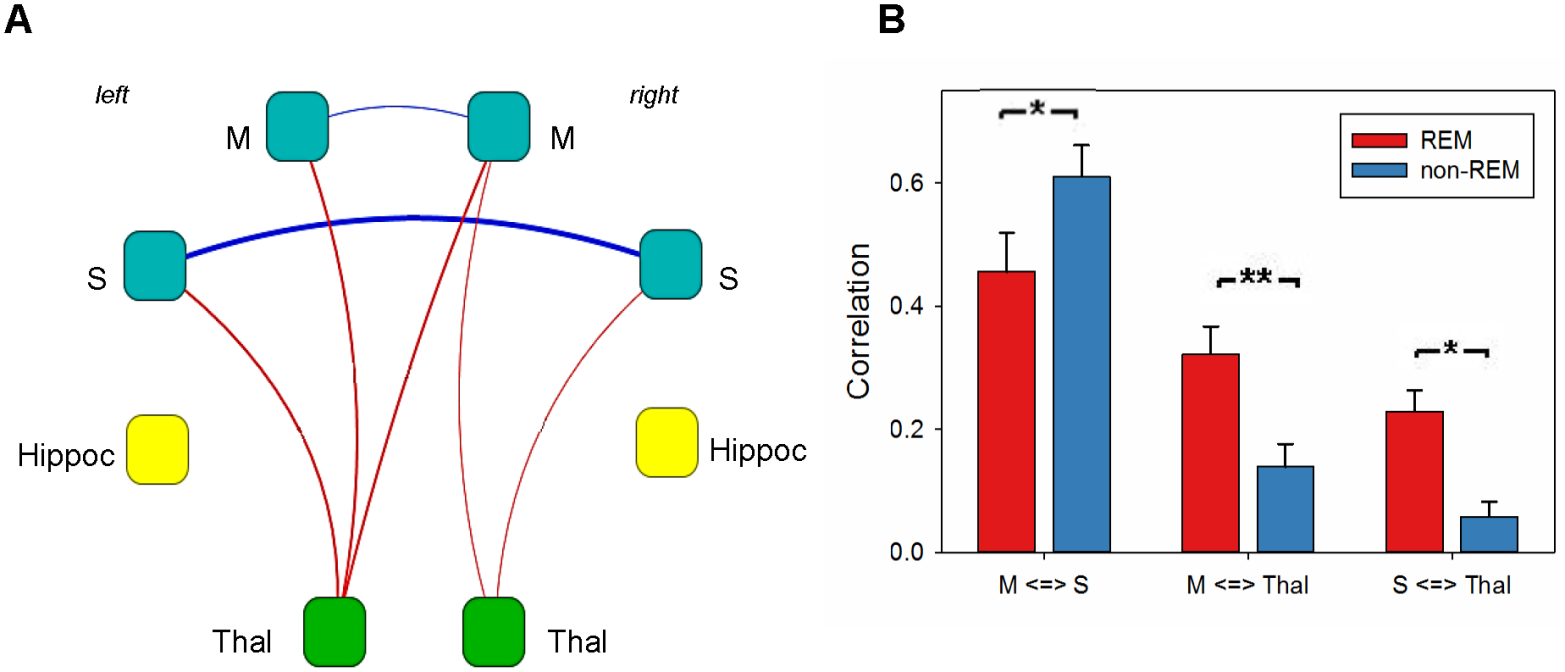
Differences in functional connectivity between the two sleep states. A. Changes in connectivity for unilateral ROIs. Only connections with p-value less than 0.05 (two-sample t-test, FDR-corrected for multiple comparisons) are shown. Edge thickness corresponds to the value of difference between REM and NREM like state, blue color of edge indicates higher connectivity in the NREM-like state, red color indicates higher connectivity in the REM-like state. Node color corresponds to the brain region: blue is for cortical, green is for thalamic and yellow is for hippocampal. B. Changes in connectivity for bilateral ROIs. Several connections based on anatomical correspondence were chosen. The connectivity from motor cortex to somatosensory cortex was significantly increased in the NREM-like state, while the connectivity between cortical and thalamic areas was significantly increased during the REM state. The stars show significance of the change (* corresponds to p < 0.05, ** corresponds to p < 0.01). Hippoc, hippocampus; M, motor cortex; S, somatosensory cortex; Thal, thalamus.

Functional connectivity differences were assessed from Pearson correlations using a paired t-test. (Fig 4A). The results showed the significant increase in functional connectivity in cortex in the NREM-like state and from cortex to thalamus in the REM-like state, but no changes in connectivity from hippocampus to other brain regions. In addition, differences in functional connectivity for bilateral ROIs were assessed (Fig 4B).

### Topology changes between two states

**Fig 5:**
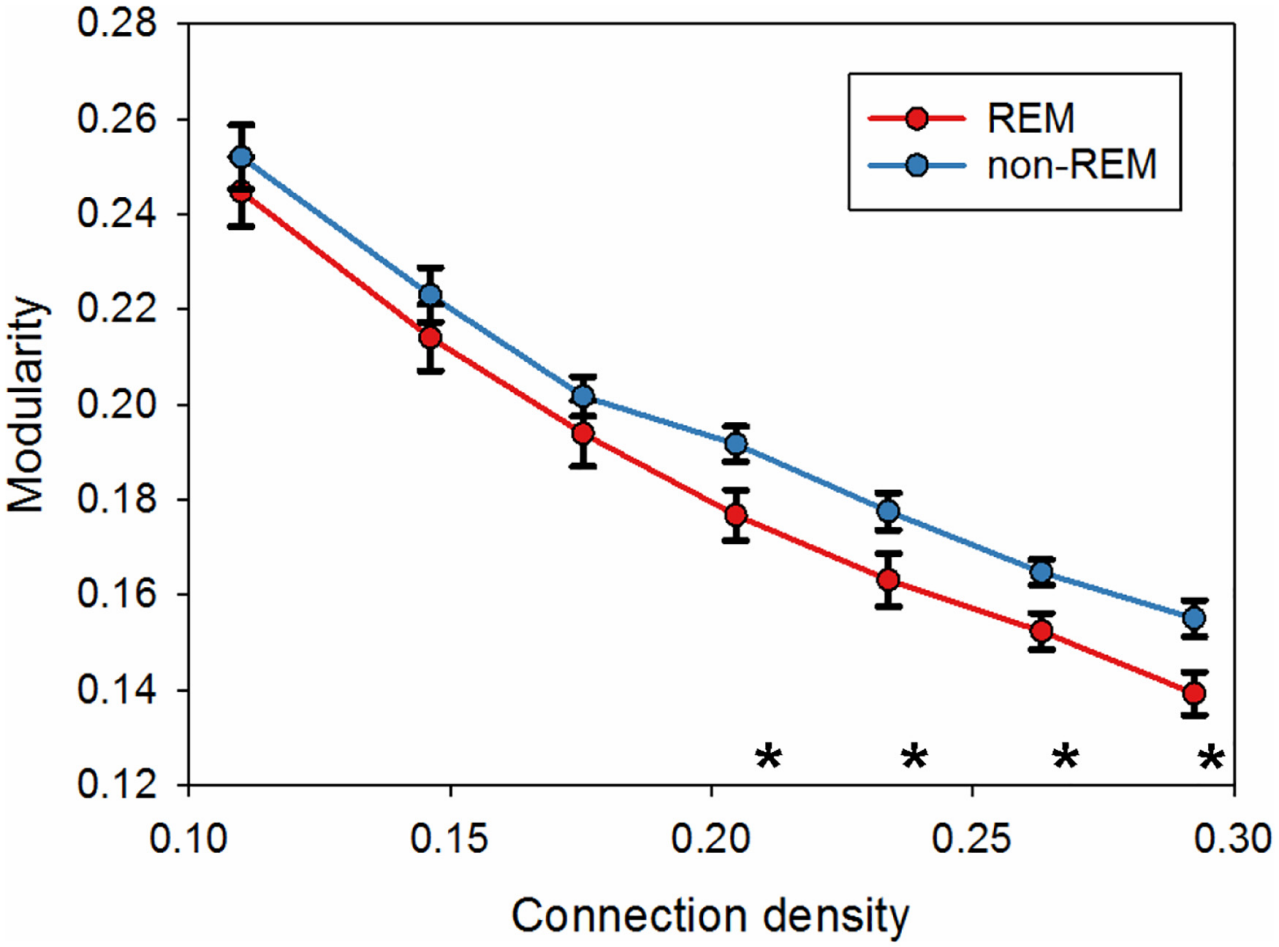
Modularity calculated for a range of different connection densities for the whole brain network. The statistical significance was tested with two-sample t-test (* p < 0.05).

The whole brain network was explored using modularity analysis. The modularity was increased in NREM-like state, when connection density was greater than 0.2 (Fig 5).

## Discussion

The purpose of this study was to estimate changes in global functional connectivity, networking and topology between sleep-like states in mechanically ventilated, urethane anesthetized rats. The major novel findings were an increase in the baseline BOLD signal in the REM-like state, increased corticocortical connectivity in the NREM-like state and increased thalamocortical connectivity in the REM-like state. In addition, modularity of the network was found to increase during the NREM-like state.

The two states were confirmed by EEG measurements in urethane anesthetized rats, similar to a previous study [17]. One of the states corresponds to the REM state in normal sleep and is characterized by decrease in slow, delta and spindle power bands, and a pronounced peak in theta band. The other state with regular high-amplitude slow activity corresponds to the NREM state in normal sleep. The striking similarity between the EEG states under urethane anesthesia and natural sleep, as well as associated changes in muscle tone, heart rate and respiration [17] suggest that urethane anesthesia in rats can serve as a model for sleep and sleep disorder studies. Opposite to the recent study [14], which was conducted in spontaneously breathing rats, we also observed clear changes in baseline BOLD signal in our ventilated rats. As duration and frequency of the “up”-states was similar to the duration of REM-states in EEG measurements, we conclude that these BOLD level states are closely associated with REM and NREM sleep states.

The REM sleep state is characterized by increased cerebral blood flow over almost all structures of the rat brain indicating increased brain metabolism [18]. If the animal is freely breathing, the autonomic functions are able to adjust breathing rate to satisfy the metabolic demands. Thus, the breathing rate is significantly higher in the REM-like state than in the NREM-like state for freely breathing urethane anesthetized rats [17]. If the rate and tidal volume of breathing is fixed with mechanical ventilation, the pCO_2_ in the circulation slightly increases with the increased metabolism, leading to vasodilation and increased BOLD signal. This allows the monitoring of sleep stages without simultaneous EEG recording. The use of mechanical ventilation also improves quality of MRI experiments because with paralyzed animals movement artifacts can be avoided.

Identification of two clearly distinct brain states lasting for several minutes allowed us to assess whether these states also possess different functional connectivity pattern in the entire brain. Since we did not have awake state as a reference, we can only discuss relative changes between the REM-like and NREM-like states. Functional corticocortical connectivity was higher during the NREM-like, whereas thalamocortical connectivity was stronger during the REM-like state. Our finding of higher corticocortical connectivity during the NREM-like state is consistent with the only previous BOLD-fMRI study in urethane anesthetized rats, in which higher functional connectivity between piriform cortex and dorsal neocortex during the NREM-like state was reported [14]. However, Wilson and coworkers did not report thalamocortical connectivity. Our finding of reduced thalamocortical functional connectivity is consistent with the classic electrophysiological finding of Steriade and coworkers that thalamocortical projection neurons are steadily hyperpolarized during slow wave sleep and thus incapable of conveying information between thalamus and cortex [19].

BOLD-fMRI studies of functional connectivity in human subjects have mainly compared light sleep and wakefulness. Consistent with our NREM-like state findings, Spoormaker et al. [20] reported that in the transition from wakefulness to light sleep, thalamocortical connectivity was sharply reduced, while corticocortical connectivity increased. Another recent human study [21] also reported decreased thalamocortical functional connectivity in two NREM sleep states compared to wakefulness. In contrast, cortical responses to transcranial magnetic stimulation were local and stereotypic in NREM sleep, but became more widespread and differentiated during REM-sleep or wakefulness [22]. The ostensibly discrepant results may simply reflect the fact that functional connectivity measured by BOLD-signal correlation over tens of seconds has very different underlying mechanisms than evoked potentials generates in tens of milliseconds.

It is important to note that changes in arterial CO_2_ levels may also affect resting-state connectivity [23]. Usually increased arterial CO_2_ levels lead to overall decreased interhemispheric functional connectivity in humans [24]. This would be consistent with our finding of decreased corticocortical connectivity during the metabolically active REM-like state. However, a recent study in rats does not lend support to this idea since the authors found interhemispheric connections between the somatosensory cortices to be generally not dependent on arterial CO_2_ or O_2_ levels [25]. This indicates that the differences we observed, more likely originate from changes caused by sleep states themselves rather global modulation of connectivity due to arterial changes.

The topology estimation in the present study showed that modularity was increased during NREM-like state as compared with the REM-like state, which is a sign of functional segregation. The finding is consistent with a human fMRI study showing that frontoparietal networks were disintegrated in transition from wakefulness to NREM sleep and were replaced by local submodules [26]. In agreement with this, a human EEG study found the cortical modularity to increase in deep sleep compared to awake state [27]. The data are also consistent with another study [28], showing the modification of brain hierarchical organization. There was an increase in both within-system and between-system integration, with within-system integration being larger than between-system integration, leading to an increase in the modularity. Furthermore, the above mentioned study combining transcranial magnetic stimulation with EEG also found higher modularity during NREM sleep as compared with REM-sleep or awake state [22]. As the arousal level is almost the same in the REM and awake states, these findings suggest that modularity increases with the decrease of arousal level.

One obvious shortcoming of the present study was the relatively large slice thickness (1.5 mm) compared to the size of the rat brain. Therefore, the number of subareas in our analysis was substantially lower than in human studies, which reduces statistical power. In addition, we could assess thalamocortical connectivity only for the thalamus as a whole. Since the anatomical organization of the thalamus is highly modular, with each thalamic nucleus communicating with a delineated segment of the neocortex and little cross-talk between the individual nuclei, one would assume thalamocortical functional connectivity to be also more segregated. Indeed, a recent fMRI study that delved into thalamic subnuclei confirmed that sleep state specific changes in thalamocortical connectivity may differ between the modules [29]. Thus, the present observation of increased thalamocortical connectivity during the REM-like state under urethane anesthesia should be interpreted with caution. Additional investigations to confirm this finding will be important; advancements of the BOLD-fMRI technique to enable higher spatial resolution will allow these experiments to take place in the future.

## Conclusion

We demonstrated that sleep-state associated changes in functional connectivity can be detected by resting-state BOLD fMRI in urethane-anesthetized rats. Importantly, these changes were in accordance with known connectivity changes between awake or REM and NREM state in human subjects. This opens up the possibility to study changes in sleep states using rodent models in future, which may shed light into the early phase of neurodegenerative diseases in which sleep abnormalities are often one of the first symptoms of the progressive disease.
